# Immunogenicity of Simulated PCECV Postexposure Booster Doses 1, 3, and 5 Years after 2-Dose and 3-Dose Primary Rabies Vaccination in Schoolchildren

**DOI:** 10.4061/2011/403201

**Published:** 2011-07-07

**Authors:** Thavatchai Kamoltham, Wiravan Thinyounyong, Pakamatz Khawplod, Phran Phraisuwan, Phana Phongchamnaphai, Gerlind Anders, Claudius Malerczyk

**Affiliations:** ^1^Office of Permanent Secretary, Ministry of Health, Nonthaburi 11120, Thailand; ^2^Provincial Health Office of Phetchabun, Ministry of Health, Phetchabun 67160, Thailand; ^3^Queen Saovabha Memorial Institute, Thai Red Cross Society and Department of Medicine, Chulalongkorn Hospital, 1871 Rama 4 Road, Bangkok 10330, Thailand; ^4^Novartis Vaccines and Diagnostics GmbH, Medical Affairs, Emil-von-Behring-Str. 76, Marburg 35041, Germany

## Abstract

*Objectives*. To assess the immunogenicity of intradermal (ID) booster doses of Purified Chick Embryo Cell rabies vaccine (PCECV, Rabipur) administered to Thai schoolchildren one, three and five years after a primary ID pre-exposure (PrEP) vaccination series. *Methods*. In this follow-up study of a randomized, open-label, phase II clinical trial, two simulated post-exposure booster doses of PCECV were administered on days 0 and 3 intradermally to 703 healthy schoolchildren, one, three or five years after primary vaccination with either two or three ID doses of 0.1 mL PCECV. Blood was drawn immediately before and 7, 14 and 365 days after the first booster dose to determine rabies virus neutralizing antibody (RVNA) concentrations. *Results*. An anamnestic response of approximately 30-fold increase in RVNA concentrations was demonstrated within 14 days after booster. All children (100%) developed adequate RVNA concentrations above 0.5 IU/mL. No vaccine related serious adverse events were seen in any of the vaccinees. *Conclusion*. ID rabies PrEP with PCECV is safe and immunogenic in schoolchildren and the anamnestic response to a two booster dose vaccination series was found to be adequate one, three, and five years after a two- or three-dose primary PrEP vaccination series.

## 1. Introduction

Rabies post-exposure prophylaxis (PEP) after an exposure to a rabid animal has been demonstrated to be efficacious using tissue culture vaccines (TCV) including purified chick embryo cell vaccine (PCECV), administered either intramuscularly (IM) or intradermally (ID) [1, 2]. However, human rabies remains a significant health problem in countries of Asia and Africa, where more than 99% of the exposures come from rabies-infected dogs that inhabit rural and urban areas. The vast majority of the estimated 55,000 human deaths that occur worldwide every year occur on these two continents [3, 4], mainly due to lack of awareness that results in delayed, inadequate PEP, or even no PEP administered to patients exposed to rabid animals. A significant number of bite exposures and rabies cases occur in children under 15 years of age [5–8]. It has been reported that in Thailand by the age of 15 years approximately one-third of all children will have experienced a dog bite, indicating the potential risk for children to be exposed to a rabid animal [9]. While PEP clearly saves lives, human rabies cases, especially in children, continue to occur despite the availability of vaccines and biologicals. Almost all of these human rabies cases could have been prevented, and almost all occurred due to a lack of receiving PEP. One possible alternative to making sure that every child received adequate PEP after exposure is to administer pre-exposure prophylaxis (PrEP) to those living in high-risk regions. The use of PrEP in children living in areas of high risk of exposure to rabies would reduce the number of vaccine booster doses required and eliminate the need to administer rabies immunoglobulin (RIG) after an exposure has occurred. For example, persons that have been vaccinated previously with a tissue culture rabies vaccine and are subsequently exposed to a rabid animal only require two booster doses of vaccine, administered on days 0 and 3, either IM or ID [4]. Previous reports have demonstrated that PCECV is immunogenic and safe when given intradermally [10–12]. Recent studies from Thailand and India revealed that the current WHO PrEP recommendations of three IM or ID doses are adequate in schoolchildren [13, 14] and toddlers [15]. A study using PCECV in toddlers administered concomitantly with Japanese encephalitis vaccine (JEV) demonstrated adequate tolerability and immunogenicity of both vaccines and indicated the suitability of introducing rabies vaccine into the Expanded Program on Immunization (EPI) schedule. In addition, a study with purified verocell rabies vaccine (PVRV) was conducted in infants, indicating adequate immune responses when rabies vaccine was administered concomitantly with pediatric routine combination vaccine (diphtheria, tetanus, whole cell pertussis, inactivated poliomyelitis; DTP-IPV) [16]. However, when infant or pre-school rabies vaccinations are missed, vaccination in early school-age children could be a practical and efficient solution to protect this most vulnerable population against rabies. In this study we investigated whether two or three ID doses of PCECV would be immunogenic in children and concluded that the current recommendation of three doses given ID is appropriate [13]. The study population, clinical trial design, and results of the primary vaccination have been published earlier [13].

## 2. Methods

### 2.1. Clinical Trial

**Figure 1:**
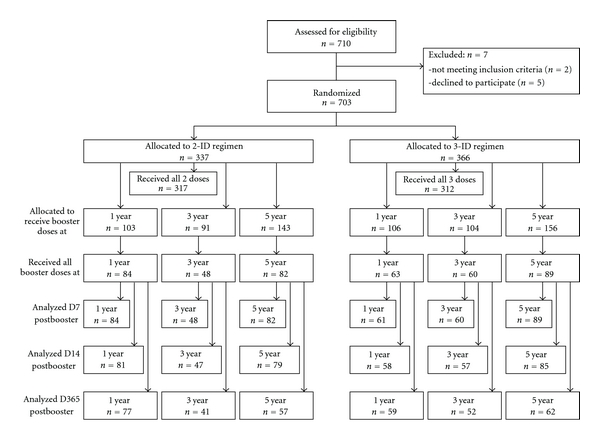
Flow diagram of study participants (according to CONSORT Statement).

In this long-term followup, the anamnestic response of Thai schoolchildren that received two (simulated) post-exposure booster doses of PCECV was investigated up to five years after the primary vaccination PrEP series was administered. Details of the study conduct have been described earlier [13]. Briefly, subjects enrolled in the clinical trial included healthy schoolchildren, aged 5 to 8 at the time that the primary vaccination with two or three 0.1 mL ID doses of PCECV was administered. Subjects were followed for one, three, or five years after primary PrEP and then received two ID booster doses of 0.1 mL PCECV on days 0 and 3, simulating the current recommended PEP booster recommendations, that is, administering the 2-dose booster doses, without RIG, as if an exposure had occurred. The PCECV used for the primary vaccination series and for the 1-year and 3-year booster doses was Novartis Vaccines' Rabipur, produced in India; batch no. 725 (potency 7.25 IU/mL). For the 5-year group, batch no. 1471 (potency 9.81 IU/mL) was used. The objectives of the study were to demonstrate long-term postbooster rabies virus-neutralizing antibody (RVNA) protection, defined as RFFIT antibody concentrations ≥0.5 IU/mL, one, three, and five years after the primary vaccination, to evaluate whether adequate RVNA concentrations is achieved in all subjects and to compare the immune responses of the 2-dose versus 3-dose ID regimen of PCECV. This study was conducted under the auspices of the Ministry of Public Health, Thailand, following the research principles set out in the Declaration of Helsinki and Good Clinical Practice guidelines. Approval of the study protocol was obtained by the Ethical Review Committee for Research in Human Subjects, Ministry of Health; all parents and legal guardians of subjects were informed of the study protocol prior to enrollment, and written informed consent was obtained from parents or legal guardians of all subjects prior to enrollment. The study was registered at ClinicalTrials.gov (identifier: NCT01107275). A flow diagram of study participants as suggested in the CONSORT Statement is given in Figure 1.

### 2.2. Serology

Blood was drawn before administration of the first of two booster doses and on days 7, 14, and 365 days later. Serology testing was performed in the same laboratory as in the first part of the study, (Queen Saovabha Memorial Institute, Bangkok, Thailand) for determination of RVNA concentrations, using the rapid fluorescent focus inhibition test (RFFIT), as described earlier [17].

## 3. Results

**Figure 2:**
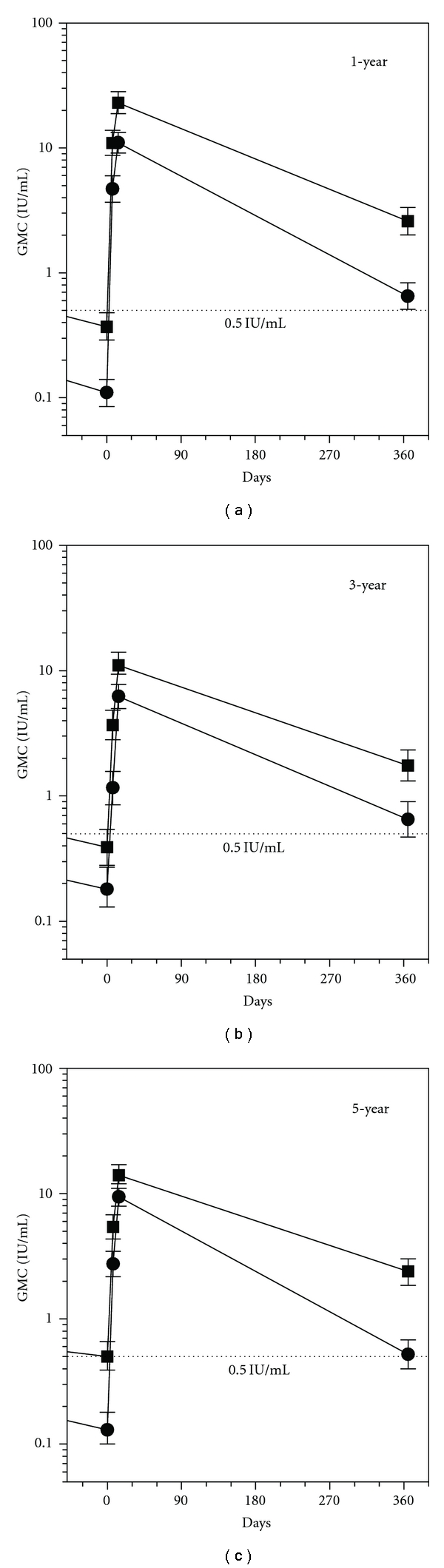
Immune response after two simulated post-exposure intradermal 0.1 mL booster doses of PCECV on days 0 and 3, administered one (a), three (b), or five years (c) after completion of a primary vaccination series. *⚫*: 2 ID doses; ■: 3 ID doses; error bars represent 95% confidence intervals ⋯: RVNA concentrations regarded as adequate for protection. (0.5 IU/mL).

One year after the primary vaccination, RVNA concentrations had decreased (Figure 2(a)) with 7% and 35% of the vaccinees still having adequate RVNA concentrations above 0.5 IU/mL, in the 2-dose and 3-dose group, respectively, (Table 1). This percentage of subjects with adequate RVNA concentrations did not change significantly over time (Figures 2(b) and 2(c)); 8% and 40% of subjects in the 3-year group and 12% and 46% of subjects in the 5-year group, respectively, maintained adequate RVNA concentrations (Table 1). After receiving two booster doses of PCECV, on day 0 and day 3, RVNA concentrations increased significantly in all study groups, thus eliciting adequate RVNA concentrations on day 7 postbooster in 100%, 97%, and 99% of the children in the 3-dose groups, and 96%, 73%, and 91% of the children in the 2-dose group, at one, three, and five years after primary vaccination, respectively. By day 14, every child (100%) had reached adequate RVNA concentrations, regardless of the time interval between primary vaccination and booster or whether having received two or three primary doses (Table 1). Thus the objective was met to demonstrate long-term postbooster RVNA protection, defined as RFFIT antibody concentrations ≥0.5 IU/mL, 1, 3, and 5 years after the primary vaccination, as well as to demonstrate that adequate RVNA concentrations are achieved in all subjects. Fourteen days after booster, the 2-dose regimen proved equivalent to the 3-dose regimen in eliciting adequate response (100% adequate RVNA concentrations in all groups), while on day 7 after booster, the percentage was lower in the 2-dose group. When comparing actual RVNA concentrations, GMCs were about 3-fold higher in the 3-dose group than in the 2-dose group. This difference was seen throughout the study (Figure 2).

## 4. Discussion

When a person has been previously immunized with a PrEP series of three doses of rabies vaccine, the current recommendations for PEP include the administration of two booster doses of a WHO-recommended tissue culture vaccine. It is neither necessary nor recommended to administer RIG to individuals that have received a tissue culture vaccine previously. The question as to whether the time interval between primary vaccination series and the PEP booster series following an exposure has an influence on the ability of a patient to elicit an anamnestic response is an important concern for public health officials that may be considering the use of PrEP to protect populations living in areas with a high risk of exposure to rabies. In this study we investigated the anamnestic response in subjects that had received a two booster dose series of PEP one, three, and five years after the primary PrEP immunization, and we have confirmed that an adequate and rapid immune response occurred in all subjects.

Interestingly, RVNA concentrations and the percentage of patients that produced adequate titers did not change significantly over the years. In subjects that had been vaccinated five years previously, approximately the same RVNA concentrations were observed as in subjects that had been vaccinated one and three years earlier. After the two-booster dose PEP series, a comparable immune response was observed in all subjects regardless of the time elapsed since their initial PrEP series. A more relevant consideration is how many doses were included in the initial primary vaccination series: those subjects that received a three-dose primary PrEP series had higher levels of RVNA concentrations and higher booster responses than subjects that received only a two-dose primary PrEP series (Figure 2). However, although GMTs of RVNA concentrations in the group that received a two-dose PrEP series were significantly lower throughout the study, in this group all subjects achieved adequate RVNA concentrations above 0.5 IU/mL, when two booster doses were given up to five years after primary vaccination. The fact that all subjects reached adequate RVNA concentrations by day 14, regardless of the time interval between primary series, and booster doses or the number of doses in the primary series is reassuring. However, the overall lower RVNA concentrations in the 2-dose group resulted in a lower percentage of adequate RVNA concentrations on day 7. In particular, in the 2-dose group adequate immune responses were only seen in 73% of children (3-year data), compared to 97–100% in the 3-dose groups. This leaves a vulnerable period of a few days in more than few subjects after a 2-dose primary vaccination series. Whether this would lead to treatment failure and development of rabies remains questionable. In PEP of previously unvaccinated subjects, adequate RVNA concentrations do not develop before day 14 either. Clearly here RIG is recommended to cover the lag period. However, in reality RIG is only administered in 2 to 10% of all cases, where it would be indicated [18], and treatment failures are seen extremely rarely. To be on the safe side, however, as administration of RIG is not considered necessary or recommended for previously vaccinated subjects, a 3-dose primary vaccination regimen might be considered more suitable for individual protection.

Additionally the question how to prove previous vaccination has to be discussed. It is not uncommon that children or parents forget about the vaccines that they had been given. A serologic testing may not be a suitable method for proof of earlier vaccination. Such testing may not be available everywhere, is quite expensive, and—most critically—would provide results too late for a decision whether to give booster doses without RIG or whether to start a complete series of PEP, including RIG when indicated. Therefore, a system of documentation of each vaccination in a booklet is preferred. As a matter of fact, in absence of documented proof of vaccination, a full PEP course including administration of RIG would be required.

The WHO recommends that diagnostic laboratory workers, rabies researchers, and other people at continuous risk (where rabies virus is present continuously, often in high concentrations, and where specific exposures to rabies are likely to go unrecognized) should have their serological titers evaluated every six months for the presence of RVNA and receive a single booster vaccination when their RVNA concentrations fall below 0.5 IU/mL [4]. For the general population living in endemic countries, it is sufficient to receive a routine ID booster series with 0.1 mL of PCECV without routine serology testing, which is expensive and difficult to perform. Due to the fact that immune memory is established in persons that have been vaccinated with a TCV, an anamnestic immune response is induced after a PEP-booster series using 0.1 mL of a TCV (PCECV) ID booster doses, as demonstrated in this study up to five years after completion of the primary vaccination. 

The results of this study are in line with results from another study investigating abbreviated and less doses intradermal pre-exposure vaccination schedules. In one of the study arms, Khawplod and coworkers administered two ID doses at two sites on a single visit as primary vaccination, using PCECV or PVRV. Upon two ID booster doses (Day 0 and 3) one year later, all subjects elicited anamnestic immune responses and adequate RVNA concentrations [19]. 

A striking additional finding in our study was that 12 of 703 children (1.7%) were actually exposed to rabies by potentially rabid animals during the study period. These were given appropriate PEP as predefined in the study protocol, and they were further excluded from serology analyses but were followed for a period of one year. All remained healthy during the observation period. The high number of exposures clearly shows that rabies is an endemic threat to children in Thailand.

## 5. Conclusion

While the current recommendation of PrEP vaccination consists of three doses of rabies vaccine administered ID or IM [4], a PrEP vaccination series using two or three doses of 0.1 mL PCECV administered ID is safe and immunogenic in school children, and anamnestic responses occurred in all subjects after two booster doses were administered up to five years later. This indicates that when an exposure occurs, two booster doses of vaccine administered ID three days apart may be appropriate in previously immunized persons that may have received only two initial doses of a PrEP series although three initial doses lead to higher immune responses and longer lasting protection. Reduced PrEP regimens would reduce the cost of protecting vulnerable populations against rabies and would promote better compliance, thus supporting opportunities to conduct mass PrEP rabies vaccination in children, the population most at risk of dying of this dreaded disease.

## Figures and Tables

**Table 1:** Number and percentage of children reaching adequate RVNA concentrations (≥0.5 IU/mL) after administration of simulated post-exposure booster doses, 1, 3, or 5 years after two or three primary vaccination doses, as determined by RFFIT.

Group	1-year				3-year				5-year			
	pre	D7	D14	D365	pre	D7	D14	D365	Pre	D7	D14	D365
2d	(6/84)	(81/84)	(81/81)	(51/77)	(4/48)	(35/48)	(47/47)	(24/41)	(10/82)	(75/82)	(79/79)	(29/57)
%	7%	96%	100%	66%	8%	73%	100%	59%	12%	91%	100%	51%
3d	(22/63)	(61/61)	(58/58)	(55/59)	(24/60)	(58/60)	(57/57)	(45/52)	(41/89)	(88/89)	(85/85)	(59/62)
%	35%	100%	100%	93%	40%	97%	100%	87%	46%	99%	100%	95%

2d: two-dose primary vaccination; 3d: three-dose primary vaccination; pre: before booster.
